# Cardiovascular Protective Effects of Adjunctive Alternative Medicine (*Salvia miltiorrhiza* and *Pueraria lobata*) in High-Risk Hypertension

**DOI:** 10.1155/2013/132912

**Published:** 2013-03-07

**Authors:** K. S. Woo, Thomas W. C. Yip, Ping Chook, S. K. Kwong, C. C. Szeto, June K. Y. Li, Alex W. Y. Yu, William K. F. Cheng, Thomas Y. K. Chan, K. P. Fung, P. C. Leung

**Affiliations:** ^1^Institute of Chinese Medicine, The Chinese University of Hong Kong, Hong Kong; ^2^Department of Medicine and Therapeutics, The Chinese University of Hong Kong, Hong Kong; ^3^Room 186, Science Centre South Block, School of Life Sciences, Biochemistry Programme, The Chinese University of Hong Kong, Hong Kong; ^4^Department of Medicine, Yan Chai Hospital, Hong Kong; ^5^Department of Medicine, Alice Ho Miu Ling Nethersole Hopsital, Hong Kong; ^6^School of Medical Sciences, The Chinese University of Hong Kong, Hong Kong

## Abstract

*Introduction*. Hypertension in association with diabetes (DM), renal impairment (RI), and left ventricular hypertrophy (LVH) increases the risk of future cardiovascular events. We hypothesize, traditional herbal medicines Danshen and Gegen (D&G) have beneficial effects on atherogenesis in these high-risk hypertensive subjects. *Subjects and Methods*. 90 asymptomatic hypertensive subjects associated with LVH (63.3%), DM (62.2%), or RI (30%) were randomized to receive D&G herbal capsules 1 gm/day, 2 gm/day, or identical placebo capsules in double-blind and parallel fashion for 12 months. Brachial flow-mediated dilation (endothelium-dependent dilation, FMD) and carotid intima-media thickness (IMT) were measured by ultrasound. All data were analyzed using the Statistical Package for Social Sciences in Windows 16.0. *Results*. Their mean age was 55 ± 8 years, and 74.4% were male. After 12 months of adjunctive therapies and compared with baseline, there were no significant changes in blood pressure, heart rate, hematological, glucose, and creatinine profiles in both placebo and D&G groups. FMD improved significantly during D&G (*P* = 0.0001) and less so after placebo treatment (*P* = 0.001). There was a mild but significant decrease in carotid IMT after D&G (*P* < 0.001) but no significant changes after placebo. A trend of better improvement in FMD after higher versus lower D&G dosages was seen. D&G were well tolerated, with no significant adverse events or blood biochemistry changes. *Conclusion*. D&G adjunctive treatment was well tolerated and significantly improved atherogenesis in high-risk hypertensive patients, with potential in primary atherosclerosis prevention.

## 1. Introduction

Atherosclerosis disease (particularly stroke and coronary artery disease) is the most important health issue in modernized society, and hypertension is an important predisposing factor [1, 2]. Hypertensive subjects with subclinical target organ damage or cardiovascular risk factors, including left ventricular hypertrophy (LVH), diabetes mellitus, or impaired renal function, are particularly vulnerable to these atherosclerotic complications in spite of best available antihypertensive therapies [3–6]. Accordingly, adjunctive primary preventive strategies are mandatory to improve their long-term natural history. 

“Danshen” (*丹參*) and “gegen” (*葛根*) (D&G) are commonly used herbal materia medica in treatment of cardiac symptoms and atherosclerosis-related disorders [7]. “Danshen” consists of the dried root and rhizome of the perennial herb *Salvia miltiorrhiza* Bge. “Gegen” is the dried roots of *Pueraria lobata* (willd.) [8]. Our preliminary works have confirmed the favorable effects of “Danshen and Gegen” on some metabolic indices and atherogenic process, which can be ascribed to their antithrombotic, lipid-modulating, antioxidant, anti-inflammatory, and phytoestrogenic properties [9–12]. A pilot double-blind placebo control secondary prevention study, on patients with coronary artery disease, has documented a significant improvement in brachial arterial endothelial function and carotid intima-media thickness, as surrogate atherosclerosis endpoints, after combination formula of D&G compared with placebo treatment [8]. Adjunctive treatment with D&G on top of standard cardiac drugs including statins, *β* blockers, aspirin, ACE inhibitors, and diuretics has been well tolerated, with no significant adverse events [8].

While the evidence base for long-term benefit of D&G treatment on hard clinical data for coronary patients is awaited with much interest, the extension of this novel but promising cardiovascular protective adjunctive regimen for primary atherosclerosis prevention in high- and very high-risk hypertensive cohort will be of tremendous clinical interests. This study proposed to evaluate the impact of D&G adjunctive therapy on top of antihypertensive treatment on improving surrogate atherosclerosis endpoints as a primary preventive strategy in high- and very high-risk hypertensive subject.

## 2. Patients and Methods

This proposed study is a double-blind, randomized, parallel, and placebo-controlled trial.

### 2.1. Subjects

90 patients with essential hypertension (SBP 160/90 mmHg before treatment) attending the hypertension clinics of the Prince of Wales Hospital, Yan Chai Hospital, and Alice Ho Miu Ling Nethersole Hospital were studied. They were associated with left ventricular hypertrophy on electrographic (ECG) or echocardiographic criteria in 57 (63.3%) patients, diabetes mellitus (fasting blood glucose >7.0 mmol/L or receiving diabetic drugs) in 56 (62.2%), patients and mild-to-moderate renal impairment (serum creatinine 120–250 *μ*mol/L) in 27 (30%) patients. Their blood pressures were currently under good control (BP 110/60 to 140/90 mmHg), and those patients with secondary hypertension, bleeding disorders, significant coexisting hepatic or gastrointestinal diseases, or on long-term anticoagulants were excluded. None has had any coronary, stroke, or other vascular events, and none was taking regular vitamins or other herbal medicines. 

### 2.2. Study Protocol

**Figure 1:**
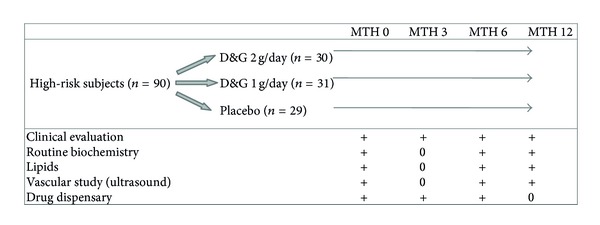
Danshen and Gegen Supplementation Protocol.

After written informed consent and successful completion of screening, all subjects were randomized by computer using a stratified block method to receive either oral D&G capsules 1 g/day, D&G capsules 2 g/day, or image-matched placebos in double-blind parallel fashion (Figure 1). Subjects, clinical staff and investigators were masked regarding the assigned treatments. Subjects were reviewed at baseline, 6, and 12 months. On each occasion, all subjects attended after 14 hours fast (except for their usual study medications) for a blood test (routine hematological, fasting glucose, lipid profile, renal, and liver functions), measurement of resting sitting blood pressure, and ultrasonic vascular study. On the assumption of baseline FMD being 7.0 ± 1.5%, enrolment of 90 patients would be adequately powered to detect a 10% relative change in FMD in post-D&G treatment (power = 80%, *α* = 0.05).

The study protocol was approved by the Institutional Ethics Committee on routine hematological human research of The Chinese University of Hong Kong, in compliance with the Declaration of Helsinki (1964). The experiment was conducted with the full understanding and consents of the subjects.

### 2.3. Vascular Studies

 The ultrasound method for measuring brachial flow-mediated dilation (FMD) and nitroglycerin-induced dilation (NTG) was performed as described by Celermajer and Deanfield [13, 14]. In brief, the diameter of the brachial artery was measured (digital caliper manually) by high-resolution B-mode ultrasound (7.5 MHz median frequency linear array L10-5 transducer and standard Advanced Technology Laboratories 5000 system) at rest, in response to reactive hyperemia, and again after sublingual nitroglycerin (200 *μ*g) administered 15 minutes after reactive hyperemia. Reactive hyperemia was induced by inflation of a pneumatic tourniquet placed around the forearm (distal to the segment of the artery being scanned) to a pressure of 220–240 mmHg for 4.5 min, followed by a release. Vessel diameter during systole was measured at a single time point 50–60 seconds post cuff deflation. FMD was expressed as vessel diameter during reactive hyperemia minus vessel diameter at baseline, over vessel diameter at baseline ×100%. Doppler-derived arterial flow (Doppler velocity time integral × vessel diameter × heart rate) was measured at rest, during reactive hyperemia, and 5 minutes after 200 *μ*g sublingual nitroglycerin. Physiologically, increased blood flow stimulates the release of vasodilators from the endothelium, such as nitric oxide, which in turn causes arterial dilation FMD. By contrast, NTG acts directly on the arterial smooth muscle and induces endothelium-independent dilation. The experiments were conducted in quiet environment, and no significant changes in their heart rate and blood pressure were observed. All scans were recorded on super-VHS videotape for subsequent offline analysis, by the same investigator (CP), blinded to subjects' identity and stage of experiment. FMD correlates significantly well with both the coronary endothelial function in the same patient [15, 16] and with extent of coronary atherosclerosis [17]. The accuracy, reproducibility, and low interobserver error for this measurement of arterial physiology have been demonstrated previously [18], which we have achieved in our previous experiments (a mean relative difference of 3% in FMD over time) [19, 20]. 

All carotid scans were performed by a single operator (CP) after a predetermined and standardized scanning protocol for the right and left carotid arteries as described by Salonen and Bots et al. [21, 22], using images of the far wall of the distal 10 mm of the common carotid arteries. All scans were recorded on super-VHS videotape for subsequent offline measurement of intima-media thickness (IMT) by a blinded investigator, using a verified automatic edge-detecting and measurement software package as we described previously [23]. The intraobserver variability for mean carotid IMT was 0.03 + 0.01 mm (coefficient of variation 1.0%).

### 2.4. Statistical Analysis

 We used the Statistical Package for Social Sciences (SPSS) 16.0 for Windows in the statistical analysis of the data. Descriptive data were expressed as mean ± SD. Collected data were evaluated using an analysis of covariance (ANCOVA) model, with baseline parameter included as a covariate. The primary efficacy endpoints were brachial FMD and carotid IMT. Differences in the clinical and vascular parameters among the 3 periods were determined using repeated measures analysis of variance (ANOVA) and compared after the Bonferroni adjustment for multiple comparison [24]. Backward stepwise multivariate analysis of variance (MANOVA) was carried out to assess the major determinants of the pooled brachial FMD and carotid IMT data at all time frames, including age, systolic and diastolic blood pressure, heart rate, hemoglobin A1-C and LDL cholesterol and creatinine levels. Statistical significance was inferred at a two-tailed *P* value of <0.05.

## 3. Results 

Their mean age was 55 + 8 years, and 67 patients (74.4%) were male. The 3 groups were fairly identical in their baseline demographic, clinical, and vascular parameters (Table 1), except there was a slightly higher diastolic blood pressure in combined D&G group (*P* = 0.017). After 12 months and compared with baseline, there were no significant changes in their blood pressures, heart rate, blood lipids, hemoglobin A1-C, and creatinine levels in all 3 groups (Table 2). 

Improvement in brachial FMD but not NTG was witnessed in both D&G (*P* < 0.0001) and placebo groups (*P* = 0.001) (Tables 2 and 3) but was more impressive after D&G treatment at 6 months (increased by 23.2%), and at 12 months (increased by 30.2%) compared with placebo treatment at 6 months (increased by 10.0%, *P* = 0.031) and 12 months, respectively (increased by 15.5%, *P* = 0.028) (Table 4). This improvement in FMD was greater after higher dosage (2 gm) D&G at 6 months (increased by 24.7%) and 12 months (increased by 32.2%) compared with lower (1 gm) D&G treatment (increased by 21.1%, *P* = 0.394 and 27.8%, *P* = 0.507, resp.) and placebo treatment (increased by 10% at 6 months *P* = 0.063 and by 15.5% at 12 months, *P* = 0.05) (Table 4). 

Carotid IMT improved significantly after both D&G (1 gm) (0.79 + 0.20 to 0.74 + 0.16 mm, decrease of 3.4%, *P* = 0.001) and D&G (2 gm) treatment (0.85 + 0.16 to 0.82 + 0.15 mm, decrease of 4.1%, *P* < 0.0001) but not after placebo (0.80 + 0.18 mm to 0.81 + 0.17 mm, increase of 1.3%, *P* = 0.510) (Table 3). On multivariate backward stepwise regression, improvement in carotid IMT was related to D&G treatment (*β* = 0.379, *P* = 0.006) and baseline LDL cholesterol (*β* = 0.276, *P* value =0.04), after adjustment for age, gender, blood pressure, hemoglobin A1-C, and creatinine level (*R* ^2^ = 0.161, *F* value =5.815, *P* < 0.005).

D&G herbal drugs were well tolerated in 3 groups, with no significant adverse events, nor any significant changes in their liver enzymes and hematological profiles (Table 5).

## 4. Discussion

Randomized trials on adjunctive traditional Chinese medicine versus placebo on cardiovascular disease are scarce. The present study is among the first ones comparing head-to-head with placebo on top of standard antihypertensive drugs. Our results of D&G adjunctive therapy on surrogate vascular endpoints in high-risk but asymptomatic hypertensive patients are very encouraging, confirming higher dose of D&G treatment is well tolerated and perhaps better improves brachial FMD, as well as regression of carotid IMT compared with placebo. Our present study however has not been adequately powered for the differential effects on FMD and IMT between the 2 D&G dosages. Both brachial FMD and carotid IMT are surrogate atherosclerotic markers predictive of stroke and cardiovascular outcome [17, 25–27]. A 0.1 mm difference in carotid IMT is associated with 1.15 (1.12 to 1.87) relative risk of myocardial infarction and 1.18 (1.16 to 1.21) relative risk of stroke [26]. These findings concur with the vascular protecting effect of D&G in patients with coronary artery disease previously reported [8].

Endothelial dysfunction (impaired FMD), oxidation of circulating LDL-cholesterol and the inward migration of oxidized LDL-cholesterol-lasden monocytes, other inflammatory infiltration, in the blood vessel wall, and subsequently intima-media thickening are the critical processes in the development of atherosclerosis [28]. Several mechanisms may explain the improvement in vascular function and structure after D&G therapy, including their lipid-lowering, antioxidant and nitric oxide production or facilitating effects, as well as their phytoestrogenic properties [8, 29–33]. The potent antioxidation property of *Pueraria lobata* isoflavones has been proposed from previous *in vitro* studies, lending support to the present findings [9, 34]. An *in vitro* endothelial and monocyte cell line experiment has revealed that D&G combination inhibits dose-dependently macrophage/foam cell transformation from fat-fed monocytes [35]. This cell-modulating mechanism underlines the possible scientific basis of our favourable vascular protective effect in primary atherosclerosis prevention in hypertensive subjects. Recently endothelial progenitor (stem) cell (EPC) activity has emerged as an innovative basic scientific concept and key process in the wear and healing of arterial endothelial cells and henceforth in vascular protection [36, 37]. However, our preliminary substudy, to evaluate the impact of D&G treatment adjunctive to traditional antihypertensive therapies, failed to support such novel mechanism of D&G treatment in vascular protection [38]. 

Earlier studies of Danshen (*Salvia miltiorrhiza*) have focused on the alcohol soluble constituents, but more recent studies targeted on hydrophilic compounds [31, 32]. Over 50 components have been isolated and identified from the extracts of danshen, including diterpene compounds, Danshensu, tanshinone 1, tanshinone IIA, sodium-tanshinone. IIA sulfonate salvianolic acid, and other phenolic acids, baicalin, *β*-sitosterol ursolic acid, daucosterol, and dimethoxy flavanone [31]. Extraction of *Pueraria lobata* (gegen) compounds, commonly called Yega, yields over 15 compounds, including puerarin, daidzein, daidzin, and other phytoestrogenic compound [33, 34]. Many of these compounds have shown antiatherogenic and favorable hemodynamic effects, either alone or in combination in tissue models experiments [9, 10, 27, 31–34]. 

Ischemia-reperfusion tissue model experiment has confirmed the efficacy of “Danshen and Gegen” combination in the ratio of 7 : 3 of the raw herbs [11, 12]. On this basis, our group has successfully produced a quality preparation of this combination (in 500 mg capsule), through several tedious processes, including the use of DNA finger printing and chemical assays for quality control and authentication, bacterial, heavy metal, and chemical toxicology monitoring, processing and aqueous extraction of raw herbs according to the guidelines of Good Manufacturing Practice (GMP) [8].

For over 50 years, the use of Danshen products has been associated with extreme safety, with no major adverse effects reported [31, 32]. The present study reiterates the high tolerability profile of combination D&G, even on top of standard drugs in coronary patients and as adjunct to standard antihypertensive therapies in high-risk hypertensive. There have been reports of interactions with warfarin, salicylate, diazepam, and ginseng [31, 32, 39–44]. Caution should be executed in clinical practice until these issues could be further clarified with wider utilization. Therapeutic (*in vitro* and *in vivo*) efficacy of individual Danshen component has been reported over the past 5 decades [45–48]. For long time, empirical clinical observations with herbal medicine suspect that these therapeutic effects are better with multiple drugs combination versus single drug. The unique and strong point of the present study is the application of a Danshen-Gegen combination formula rather than single herbal drug component, which was found to be safe and effective in improving early stage of atherogenesis. 

The present study documents an improvement in brachial FMD and carotid IMT after a moderate dosage (1-2 g/day) of D&G therapy, independent of blood pressure or lipid lowering. It is quite possible that lower doses of D&G therapy and over longer period might result in similar or better benefit. Further study will be required to address specifically the dose response and different combination formulae issues, as well as drug interaction with standard antihypertensive drugs, which conceivably will require another prospectively planned study and much bigger numbers of subjects. The possible improvement in carotid IMT (4%) and FMD (30%), as surrogate atherosclerosis markers observed over 12 months intervention in this pilot project, although statistically significant, is quite subtle and may be of borderline biological significance. Nevertheless, together with our previous work in coronary patients, the present study with encouraging vascular protecting findings and safety profiles will provide the much needed evidence base for the application of adjunctive complementary medicine for both primary and secondary atherosclerosis prevention. Longer intervention studies focusing on hard clinical endpoints, including stroke, heart attacks, and total mortality, are awaited with enthusiasm and great interest. 

## 5. Conclusion

Asymptomatic essential hypertensive subjects with LVH, DM, or renal impairment have a greater atherosclerosis burden than subjects with hypertension only. Danshen and Gegen adjunctive treatment has been well tolerated and significantly improved atherogenic process in these high-risk hypertensive patients, with potential in primary prevention of atherosclerosis.

## Figures and Tables

**Table 1:** Baseline characteristics of 90 hypertensive subjects.

Item	Placebo^1^	D&G (1 gm)	D&G (2 gm)	D&G (1 gm + 2 gm)^2^	*P* value	*P* value
	(*n* = 29)	(*n* = 31)	(*n* = 30)	(*n* = 61)		(1 versus 2)
Age	55.6 ± 8.2	51.7 ± 7.7	56.9 ± 7.4	54.3 ± 8.0	0.031	0.484
Gender						
Male	20	27	20	47	0.134	0.445
Female	9	4	10	14		
DM	18	11	17	28	0.091	0.152
LVH	12	13	10	23	0.749	0.738
CRF	10	12	12	24	0.901	0.657
Body weight (kg)	70.8 ± 12.9	70.0 ± 11.0	69.5 ± 14.7	69.7 ± 12.8	0.920	0.703
BMI (kg/m^2^)	26.8 ± 4.8	25.8 ± 3.0	25.9 ± 4.9	25.9 ± 4.0	0.641	0.346
SBP (mmHg)	132.6 ± 18.5	128.8 ± 11.1	133.6 ± 15.9	131.2 ± 13.8	0.449	0.694
DBP (mmHg)	77.4 ± 11.2	82.4 ± 9.3	83.5 ± 9.8	82.9 ± 9.5	0.053	0.017
TC (mmol/L)	4.81 ± 0.92	5.36 ± 1.06	5.16 ± 0.94	5.26 ± 1.00	0.113	0.051
TG (mmol/L)	1.99 ± 1.37	2.53 ± 2.41	1.69 ± 0.75	1.94 ± 1.34	0.159	0.886
HBA1C (%)	7.46 ± 2.62	6.81 ± 2.04	6.41 ± 0.99	6.60 ± 1.57	0.251	0.180
Creatinine (umol/L)	110.0 ± 40.6	127.9 ± 57.9	110.1 ± 49.3	119.1 ± 54.2	0.278	0.421
FMD (%)	5.46 ± 1.27	5.38 ± 1.89	4.97 ± 1.52	5.17 ± 1.71	0.490	0.465
IMT (mm)	0.80 ± 0.18	0.79 ± 0.20	0.85 ± 0.16	0.82 ± 0.18	0.372	0.607

BMI: body mass index, CRF: chronic renal failure, DM: diabetis mellitus, DBP: diastolic blood pressure, FMD: flow-mediated dilation, HBA1-C: hemoglobin A1-C, IMT: intima-media thickness, LVH: left ventricular hypertrophy, SBP: systolic blood pressure, TC: total cholesterol, TG: triglyceride, 1: placebo, and 2: D&G (1 gm + 2 gm).

**Table 2:** Changes in clinical and vascular parameters after 12 months.

	Placebo (*n* = 29)		D&G (1 gm/day) (*n* = 31)		D&G (2 gm/day) (*n* = 30)	
	Baseline	12 months	Baseline	12 months	Baseline	12 months
SBP (mmHg)	124.4 ± 17.8	136.0 ± 24.0	132.3 ± 13.5	127.3 ± 11.0	134.9 ± 18.8	140.3 ± 14.6
DBP (mmHg)	78.7 ± 13.7	81.90 ± 12.50	88.0 ± 7.6	84.1 ± 8.3	82.9 ± 11.0	84.4 ± 7.1
TC (mmol/L)	4.8 ± 0.9	4.7 ± 1.0	5.4 ± 1.1	5.1 ± 1.0	5.2 ± 0.9	5.0 ± 0.9
TG (mmol/L)	2.0 ± 1.4	2.0 ± 1.5	2.2 ± 1.7	1.8 ± 1.3	1.7 ± 0.8	1.6 ± 0.9
HBA1C (%)	7.5 ± 2.6	7.3 ± 2.1	6.8 ± 2.0	7.0 ± 2.4	6.4 ± 1.0	6.6 ± 0.9
Creatinine (mol/L)	110.0 ± 40.6	109.4 ± 50.3	127.9 ± 58.0	130.0 ± 64.0	110.1 ± 49.3	110.4 ± 83.2
Reactive hyperemia (%)	543 ± 192	485 ± 105	615 ± 208	606 ± 154	535 ± 206	505 ± 133
FMD (%)	5.5 ± 1.3	6.2 ± 1.1*	5.4 ± 1.9	7.0 ± 2.1**	5.0 ± 1.5	6.2 ± 1.6**
NTG (%)	15.2 ± 2.5	15.9 ± 3.0	18.3 ± 11.9	16.7 ± 3.3	15.4 ± 3.3	16.1 ± 3.1
IMT (mm)	0.80 ± 0.18	0.81 ± 0.17	0.79 ± 0.20	0.74 ± 0.16**	0.85 ± 0.16	0.82 ± 0.15**

Compared with baseline within group: *P = 0.001;  **P < 0.0001. SBP: systolic blood pressure, DBP: diastolic blood pressure, TC: total cholesterol, TG: triglyceride, HBA1-C: hemoglobin A1-C, FMD: flow-mediated dilation, NTG: nitroglycerin-induced dilation, and IMT: intima-media thickness, Reactive hyperemia =  (velocity − time  integral_2_ × heart  rate_2_)/(velocity − time  integral_1_ × heart  rate_1_) × 100.

**Table 3:** Changes in vascular parameters at 6 and 12 months.

Vascular parameters	Baseline	6 months	12 months	*P* value^1^	*P* value^2^
FMD (%)					
Placebo	5.5 ± 1.3	5.9 ± 1.2	6.2 ± 1.1	0.001	0.0001
D&G (1 gm)	5.4 ± 1.9	6.3 ± 2.1	7.0 ± 2.1	<0.0001	<0.0001
D&G (2 gm)	5.0 ± 1.5	5.9 ± 1.5	6.2 ± 1.6	<0.0001	<0.0001
*P* value^3^	0.850	0.411	0.088		
*P* value^4^	0.275	0.917	0.933		
*P* value^5^	0.465	0.693	0.347		
IMT (mm)					
Placebo	0.80 ± 0.18	0.80 ± 0.17	0.81 ± 0.17	0.385	0.510
D&G (1 gm)	0.79 ± 0.20	0.77 ± 0.18	0.74 ± 0.16	0.001	0.001
D&G (2 gm)	0.85 ± 0.16	0.83 ± 0.15	0.82 ± 0.15	0.006	<0.0001
D&G (2 gm + 1 gm)	0.82 ± 0.18	0.80 ± 0.17	0.78 ± 0.16	<0.0001	<0.0001
*P* value^3^	0.850	0.457	0.130		
*P* value^4^	0.272	0.637	0.796		
*P* value^5^	0.607	0.037	0.001		
NTG (%)					
Placebo	15.2 ± 2.5	15.6 ± 2.4	15.9 ± 3.1	0.276	0.510
D&G (1 gm)	16.0 ± 2.8	16.7 ± 3.0	16.7 ± 3.3	0.376	0.914
D&G (2 gm)	15.4 ± 3.3	15.9 ± 3.2	16.1 ± 3.1	0.198	0.301
*P* value^3^	0.154	0.203	0.437		
*P* value^4^	0.901	0.684	0.882		

1: 6 months versus baseline (paired *t*-test); 2: 12 months (paired *t*-test) versus baseline; 3: D&G (1 gm) versus placebo (ANOVA); 4: D&G (2 gm) versus placebo (ANOVA); 5: combined D + G (1 gm +2 gm) versus placebo (ANOVA).

D&G: Danshen + Gegen.

FMD: flow-mediated dilation.

IMT: intima-media thickness.

NTG: nitroglycerin-induced dilation.

**Table 4:** Percent change of FMD from baseline.

Group	Baseline (mm)	6 months (%)	12 months (%)	*P* value^1^	*P* value^2^
D&G (1 gm)	5.38 ± 1.89	21.6	27.8	0.001	0.001
D&G (2 gm)	4.97 ± 1.52	24.7	32.2	0.001	0.001
D&G (2 gm + 1 gm)	5.17 ± 1.71	23.2	30.2	0.001	0.001
Placebo	5.46 ± 1.27	10.0	15.5	0.001	0.001
*P* value^3^	0.867	0.147	0.171		
*P* value^4^	0.275	0.063	0.05		
*P* value^5^	0.465	0.031	0.028		

*P* value^1^: comparison of 6 months versus baseline.

*P* value^2^: comparison of 12 months versus baseline.

*P* value^3^: comparison between D&G (1 gm) with placebo (Kruskal-Wallis test).

*P* value^4^: comparison between D&G (2 gm) with placebo (Kruskal-Wallis test).

*P* value^5^: comparison between D&G (2 gm + 1 gm) with placebo (Mann-Whitney test).

**Table 5:** Changes in hematological and biochemical parameters.

Item	Placebo		D&G (1 gm)		D&G (2 gm)	
	Baseline	12 months	Baseline	12 months	Baseline	12 months
WBC (10^9^/L)	7.34 ± 2.00	7.60 ± 1.70	7.0 ± 2.20	5.90 ± 7.0*	7.10 ± 1.80	6.70 ± 1.90
Haemoglobin (%)	14.30 ± 1.20	14.0 ± 1.40	14.50 ± 1.60	14.40 ± 1.40	14.30 ± 1.30	14.20 ± 1.60
Platelet (10^9^/L)	252 ± 54	272 ± 49	277 ± 62	271 ± 62	257 ± 65	251 ± 63
Glucose (mmol/L)	6.80 ± 2.40	6.90 ± 2.90	7.20 ± 3.10	7.20 ± 3.30	6.60 ± 1.30	6.50 ± 1.30
ALP (u/L)	74.80 ± 21.3	67.90 ± 13.80	74.60 ± 24.0	67.90 ± 18.50	75.0 ± 15.0	73.80 ± 17.80
ALT (u/L)	28.30 ± 15.20	31.30 ± 18.20	27.80 ± 14.40	24.50 ± 18.50	30.50 ± 15.80	29.90 ± 15.60

*Compared with placebo *P* = 0.017.

ALP: alkaline phosphatase.

ALT: alanine aminotransferase.

WBC: white blood cells.
